# Urotropin vs. Helmitol

**Published:** 1904-06

**Authors:** 


### Urotropin vs. Helmitol.

Erich Bruch, in a brochure entitled, Experimental Investigations of Urotropin and Urotropin Anhydromethylenecitrate (Breslau, 1903), details some comparative tests of these two drugs which he made at Prof. Stern's Royal University Clinic, Breslau. He concludes from his exhaustive investigations that the latter, which has been introduced under the names of new-urotropin and helmitol, is inferior to urotropin. As it contains only 40.7 per cent. of urotropin, it takes 1.5 gram (22½ grains) thereof to equal the action of 0.6 gram (9 grains) of urotropin. Hence in order to obtain equal results, the dose of new-urotropin or helmitol must be based on its urotropin contents, that is must be two and a half times as great. This correspondingly larger dose is not more effective than urotropin, either in normal urine or in urine containing albumin or pus.

